# American Dental Association

**Published:** 1869-06

**Authors:** J. G. Ambler

**Affiliations:** Chairman. 25 W. 23d Street, N. Y.


### ARTICLE VII.

### *American Dental Association.*

Mr. Editor:—Permit me to call the attention of the delegates to and members of the Dental Association, which holds its annual meeting at Saratoga on the first Tuesday in August, to the desire intimated and expressed by some, of making our next meeting additionally attractive and interesting, by combining the social elements with our professional gathering, and to this end the committee of arrangements would suggest and urge the delegates and members to bring their wives and daughters with them, in the hopes that by so doing additional interest will cluster around our gathering, and add much to the pleasure and gratification to ourselves and those connected with us.

The committee will see that accomodations are provided for all who will give timely notice of their wishes by addressing the chairman, stating what accomodations they require.

J. G. Ambler, Chairman.
25 W. 23d Street, N. Y.

